# Global land system maps at 1 km resolution for 1.5 °C climate

**DOI:** 10.1038/s41597-025-04991-0

**Published:** 2025-04-22

**Authors:** Yifan Gao, Haewon McJeon, Yang Ou, Li Chen, Jiaying Lv, Delin Fang, Yuanhui Wang, Sijing Ye, Changqing Song, Peichao Gao

**Affiliations:** 1 State Key Laboratory of Earth Surface Processes and Hazards Risk Governance, Faculty of Geographical Science, Beijing Normal University, Beijing, 100875 China; 2 Graduate School of Green Growth & Sustainability, Korea Advanced Institute of Science and Technology, Daejeon, Republic of Korea; 3 College of Environmental Sciences and Engineering, Peking University, Beijing, China; 4 Institute of Carbon Neutrality, Peking University, Beijing, China

**Keywords:** Geography, Environmental sciences

## Abstract

Climate pledges are a key pathway for achieving temperature control but also exert profound cascading effects on the global Earth system. Evaluating such cascading effects often requires land change forecast products. However, current forecast products are all based on shared socioeconomic pathways (SSPs) and representative concentration pathways (RCPs). We proposed a comprehensive approach to generating 2100 land system maps under the 1.5 °C climate scenario and the baseline scenario by harmonizing the Global Change Analysis Model (GCAM), Globeland30, and the Land-N2N model. The maps exhibit a spatial resolution of 1 km and consist of 30 land system types, reflecting local high, medium, and low densities of land cover types. Additionally, we evaluated the Land-N2N using the kappa coefficient and figure of merit (FoM). The average kappa coefficient and FoM values across all the basins were 83.14% and 8.48%, respectively, demonstrating the reliability of the Land-N2N model in simulating land system changes. The dataset provides finer resolution quantitative support for global climate change mitigation and essential data for related research.

## Background & Summary

Mitigating the rapid increase in temperature has become a core objective of climate pledge. In 2015, the Paris Agreement was adopted with the goal of limiting the global average temperature increase to well below 2 °C and restricting it to 1.5 °C above preindustrial levels^1^. Before the 26th Conference of Parties (COP26) in Glasgow, 154 parties had submitted new or updated their nationally determined contributions (NDCs)^2^. Analysis for the 28th Conference of Parties (COP28) concluded that the existing NDCs are insufficient to limit the temperature increase in line with the Paris Agreement^3^. Parties were encouraged to move swiftly from pledges and commitments to tangible actions at COP28^3^. In addition, the COP28 advised parties to update their NDCs by 2025^4^.

Focusing on land change under climate pledges is crucial for mitigating temperature increases. Roe, *et al*.^5^ reported that actions in the land sector can significantly contribute to achieving the 1.5 °C climate. In particular, the majority of the newly submitted or updated NDCs included land-sector mitigations^5^. For example, China’s NDC outlines measures such as vegetation restoration, land-use adjustments, natural forest protection programs, and other ecological conservation and restoration (https://unfccc.int/NDCREG). The United States of America stated that “federal and state governments will invest in forest protection and forest management, and engage in intensive efforts to reduce the scope and intensity of catastrophic wildfires, and to restore fire-damaged forest lands” (https://unfccc.int/NDCREG). The importance of land in climate pledges lies in the fact that land changes, such as the expansion of cropland, can cause the release of carbon stored in natural vegetation and soils into the atmosphere^6^. Carbon emissions caused by land change are a key factor in the increase in temperature^6,7^. Specifically, carbon emissions caused by land change are a major source of atmospheric carbon^7^, accounting for one-third of all carbon emissions caused by human activities since the Industrial Revolution^8^.

With respect to the newly submitted or updated NDCs at the COP26, Iyer, *et al*.^9^ evaluated the impact of these NDCs on the peak temperature and temperature changes in 2100 using the Global Change Analysis Model (GCAM). Specifically, Iyer, *et al*.^9^ set 27 scenarios by combining three ambition levels (NDC, NDC+, and NDC++), three decarbonization rates (2%, 5%, and 8%), and three timelines for achieving net-zero emissions (on schedule, five years earlier, and 10 years earlier). Among the 27 scenarios, 17 scenarios are able to limit the temperature increase to below 1.5 °C by 2100. The temperature changes in the remaining 10 scenarios were all greater than 1.5 °C but did not exceed 2 °C.

However, the spatially explicit impacts of these climate pledges on global land change throughout this century (until 2100) remain unclear. Existing land datasets for 2100 primarily focus on the impacts of SSPs and RCPs^10,11^, rather than on the impacts of newly submitted or updated climate pledges at the COP26. SSPs describe five distinct socioeconomic development pathways for the future trajectory of the world^12,13^. RCPs describe different levels of greenhouse gas concentrations and radiative forcing that may occur in the future^14^. For example, Zhang, *et al*.^10^ forecasted future land changes under five typical scenarios (SSP1-2.6, SSP2-4.5, SSP3-7.0, SSP4-3.4, and SSP5-8.5). Li, *et al*.^15^ forecasted future land changes under four scenarios (RCP2.6, RCP4.5, RCP6.0, and RCP8.5).

In addition, existing global land datasets for 2100 reflect changes in land use or land cover, but not in land system^10,11,15–17^. In the study, land systems refer to expansions of land use or land cover. Specifically, land systems can reflect the spatial distributions of land use or land cover, as well as local nature or social characteristics^18–22^. Compared with land systems, land use or land cover focuses on direct human utilization of land or its natural state, ignoring the local densities. For example, land use or land cover classifications include basic land types such as cropland, forest, and grassland. In this study, land systems include land types such as “Low density cropland”, “Medium density artificial surfaces”, and “High density grassland”. In existing land datasets in 2100, although Gao, *et al*.^18^ and Lv, *et al*.^20^ forecasted land system changes, the study areas were limited to the Tibetan Plateau and China. And Wang, *et al*.^23^ forecasted land system changes from 2010 to 2050, but the study areas were limited to a certain country.

In this study, we forecasted the global land system changes from 2020 to 2100 at a 1 km resolution by integrating the GCAM^9^ with the Land-N2N model^18,19^. We generated a dataset consisting of a 2010 land system map, a 2020 land system map, and 2100 land system maps under the 1.5 °C climate scenario and the baseline scenario. The land system maps refer to the land system data. First, we generated the land demands in 2100 using the GCAM under the two scenarios. Second, we simulated the land system changes from 2020 to 2100 to meet the demands generated by the GCAM. The choice of the Land-N2N model was based on its ability to establish many-to-many relationships between land types and demands, thus avoiding the need for calibration of the GCAM due to differences in land definitions between land data and the GCAM^24^. Third, we evaluated the Land-N2N model by simulating land system changes from 2010 to 2020 in each basin. The datasets provide a more comprehensive solution from a land system perspective to mitigate global temperature increases and support the adjustment of future climate pledges.

## Methods

### Overall approach

**Fig. 1:**
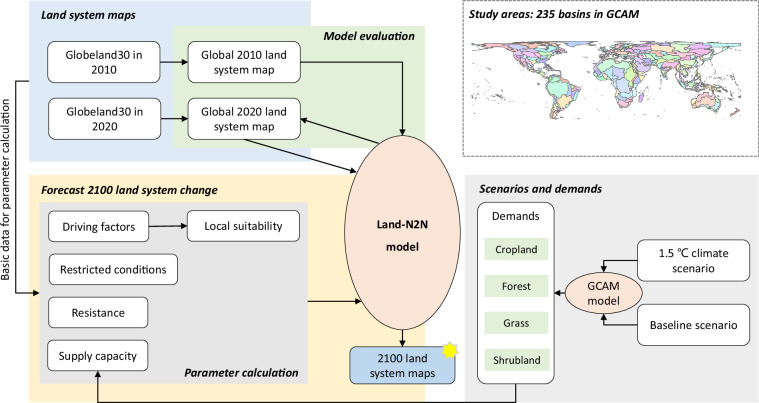
Overall approach for forecasting 2100 land system maps.

We proposed a comprehensive approach to forecasting land system changes from 2020 to 2100 by harmonizing Globeland30, the GCAM, and the Land-N2N model. The overall approach is shown in Fig. 1. Specifically, we used the basin boundaries provided by the GCAM to divide the world into 235 water basins, allowing separate forecasts and evaluations of land system changes in each basin. For each basin, we first established the global 1 km land system maps based on Globeland30 for 2010 and 2020. The 2010 and 2020 land system maps were used as the baseline maps for the forecasts and evaluations, respectively. Second, we calculated the 2100 demands using the GCAM under the 1.5 °C climate scenario^9^ and the baseline scenario^25^. The demands were used as inputs for the Land-N2N model. We regrouped the land types in the GCAM into four categories to represent land demands, namely cropland, forest, grassland, and shrubland. Third, we forecasted the future land system changes from 2020 to 2100 using the Land-N2N model^18,19^. We calculated the necessary simulation parameters, including local suitability, restricted conditions, resistance, and supply capacity. Finally, we evaluated the performance of the Land-N2N model in each basin using the kappa coefficient^26^ and figure of merit (FoM)^11^.

### Data materials

The datasets used in the study are shown in Table S1 in the Supplementary Information. The datasets consist of land cover data and driving factors. The land cover data were used to establish the land system maps for 2010 and 2020. In this study, we selected the Globeland30^27,28^ dataset (https://www.webmap.cn/commres.do?method=globeIndex) to establish land system maps for 2010 and 2020. The Globeland30 dataset provides global land cover data at a 30 m resolution and consists of ten land cover types, namely cropland, forest, grassland, shrubland, wetland, water bodies, tundra, artificial surfaces, bareland, and permanent snow and ice.

Driving factors refer to the spatial natural, social, and economic factors that influence land changes. Driving factors were used to calculate local suitability, which is a key spatial parameter for forecasting land system changes. To comprehensively capture the driving factors of all land system types, we selected eight categories of driving factors, consisting of soil, socioeconomic factors, accessibility, agriculture and vegetation, terrain, climate, livestock, and land density, for a total of 65 driving factors^18^. All the driving factors were resampled to a 1 km resolution.

### Land system maps for 2010 and 2020

**Fig. 2:**
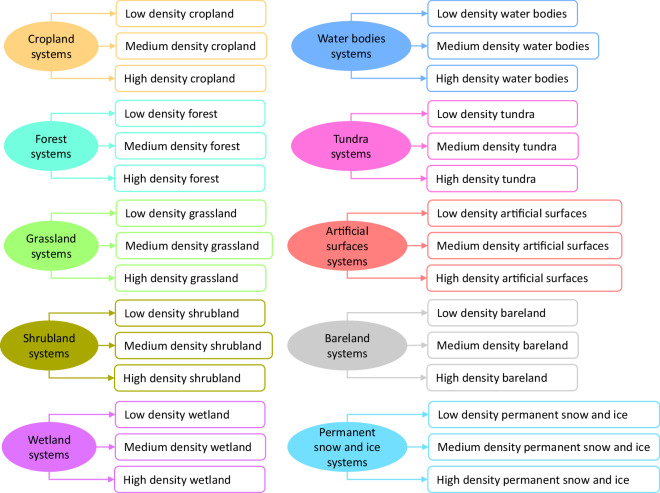
30 land system types.

We established land system maps using an upscaling approach^18,21^. We generated 30 land system types by combining ten system types with three density levels (Fig. 2), namely low, medium, and high density. There are two reasons for classifying land categories into three density levels. First, we aimed to reflect the local density of land cover as comprehensively as possible while also considering an appropriate level of global simulation complexity. Second, land system types with different density levels offer more possibilities for management approaches from a land management perspective. For example, when we want to increase cropland area, we should not only consider changing other land categories into cropland but also consider increasing the density levels of existing cropland.

**Fig. 3:**
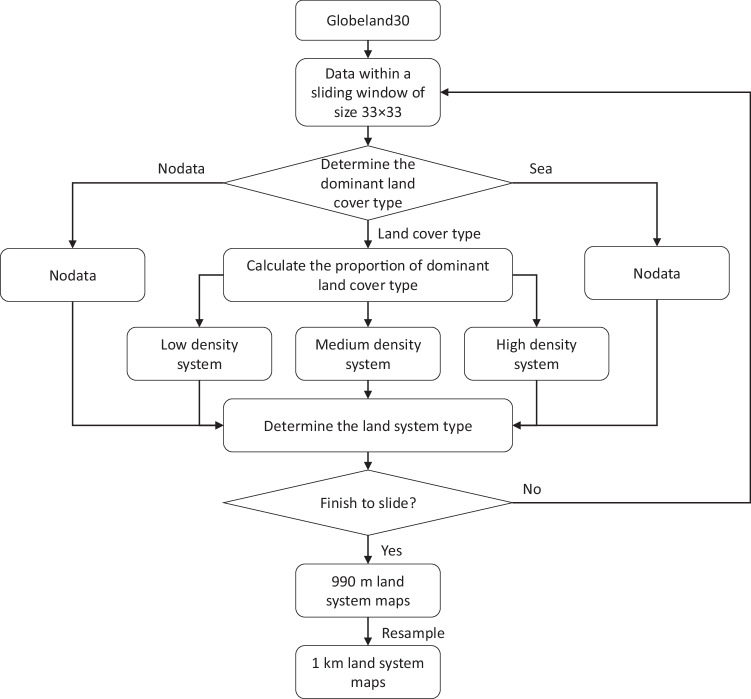
Rules for generating land system maps.

The principle for establishing land systems is to slide the Globeland30 dataset using a specified sliding window. In each sliding window, the system type was determined on the basis of the dominant land cover within the sliding window, and the density level was determined by the proportion of the dominant land cover type (Fig. 3). To determine the density level, we first calculated the global dominant proportions in each sliding window for each system type and then calculated the natural break thresholds^29^, as shown in Table 1. For example, in the case of cropland, if cropland is the dominant type within a sliding window, and its proportion ranges between 19.8% and 65.8%, the upscaled land system type for the sliding window is classified as low density cropland. If its proportion ranges between 65.8% and 87.6%, the land system is classified as medium density cropland. If its proportion exceeds 87.6%, the land system is classified as high density cropland. Here, we use “low density” as an adjective modifying the following noun, namely the dominant type. “Low density” indicates that the proportions of the dominant type are lower than in “medium density” and “high density” land systems. In addition, the reason for using natural break thresholds is that this method is commonly used for classification, as it minimizes differences within each class while maximizing differences between classes^29,30^. Density levels calculated using the natural break method can effectively reflect the characteristics of each level. In this study, we first used 33 × 33 sliding windows to generate 990 m global land system maps and then resampled the 990 m land system maps to 1 km land system maps.

**Table 1:** Natural break thresholds for classifying density levels.

Land cover type	Minimum	Natural break 1	Natural break 2
Cropland	19.8%	65.8%	87.6%
Forest	19.3%	66.8%	87.9%
Grassland	18.8%	66.7%	87.5%
Shrubland	18.8%	63.8%	85.7%
Wetland	17.7%	63.6%	86.3%
Water bodies	19.1%	62.6%	86.5%
Tundra	22.1%	70.0%	89.4%
Artificial surfaces	19.6%	61.0%	83.6%
Bareland	19.4%	68.1%	89.6%
Permanent snow and ice	22.2%	68.0%	89.4%

### Scenarios and demands

In this study, we established two scenarios, namely the baseline scenario^25^ and the 1.5 °C climate scenario^9^. Here, the baseline scenario was derived from the current policy scenario in the study of Ou, *et al*.^25^. The baseline scenario assumes that countries continue decarbonizing at the same annual decarbonization rate^25^ without implementing NDCs. For each country, the annual decarbonization rate is defined as the annual rate of the gross domestic product (GDP)^25^. According to the results of Ou, *et al*.^25^, there is an approximately 20% likelihood that the global temperature change will exceed 3 °C and an approximately 70% likelihood that it will exceed 2 °C under the baseline scenario.

The 1.5 °C climate scenario was derived from the study of Iyer, *et al*.^9^, which consists of 27 scenarios for future temperature changes. We selected the seventh scenario as the 1.5 °C climate scenario. The 1.5 °C climate scenario assumes that countries will implement NDCs as declared. Moreover, countries will implement 8% decarbonization and achieve the net-zero pledges at the target year. Under the 1.5 °C scenario, the peak temperature change before 2100 will approach 1.8 °C, but it will ultimately be controlled below 1.5 °C by 2100.

The demands for the two scenarios were based on the GCAM. The GCAM is a global equilibrium model^31^. The GCAM includes five major modules, namely water, energy, climate, socioeconomics, and land^31^. In the GCAM, the world is divided into 235 water basins, with land type areas for each basin generated at five-year intervals from 1990 to 2100^31^. Here, the area from 1990 to 2020 has already been calibrated. The land module in the GCAM consists of 70 land types^18^. We regrouped the areas of 67 land types in the GCAM into four demand categories, namely cropland, forest, grass, and shrubland, on the basis of the content of the land types (Table 2) in each water basin. We did not regroup the tundra, rock-ice-desert, and urban land types as demand categories because their areas are assumed to remain unchanged. The demand results are listed in Table S2.

**Table 2:** Four demands and their corresponding land types in the GCAM.

Demands	Land types in GCAM
Cropland	Corn_IRR/RFD_hi/lo
	FiberCrop_IRR/RFD_hi/lo
	FodderGrass_IRR/RFD_hi/lo
	FodderHerb_IRR/RFD_hi/lo
	MiscCrop_IRR/RFD_hi/lo
	OilCrop_IRR/RFD_hi/lo
	PalmFruit_IRR/RFD_hi/lo
	Rice_IRR/RFD_hi/lo
	RootTuber_IRR/RFD_hi/lo
	SugarCrop_IRR/RFD_hi/lo
	Wheat_IRR/RFD_hi/lo
	BiomassGrass_IRR/RFD_hi/lo
	BiomassTree_IRR/RFD_hi/lo
	OtherGrain_IRR/RFD_hi/lo
	OtherArableLand
Forest	Forest
	UnmanagedForest
	ProtectedUnmanagedForest
Grassland	GrassLand
	ProtectedGrassland
	Pasture
	UnmanagedPasture
	ProtectedUnmanagedPasture
Shrubland	ShrubLand
	ProtectedShrubland

Note: The GCAM incorporates irrigation types (irrigated or rainfed) and fertilizer levels (high fertilizer and low fertilizer levels) for crops. Notably, “irrigated”, “rainfed”, “high fertilizer” and “low fertilizer” are abbreviated as “IRR”, “RDF”, “hi”, and “lo”, respectively. Any combination of irrigation type and fertilizer level constitutes a land type in the GCAM.

### Forecasting the 2100 land system changes

We used the Land-N2N model^18,19,32^ to forecast the 2100 land system changes under the baseline scenario and the 1.5 °C climate scenario. The Land-N2N model, proposed by Gao, *et al*.^19^, was designed to simulate spatial explicit land system changes in response to the changes in demands. Compared with the original CLUMondo model^33,34^, the Land-N2N model is more efficient and effective at simulating land system changes^18^.

The Land-N2N model can be used to simulate spatially explicit land changes through multiple iterations to fulfill demands. Land types can be changed in each iteration. The rules for changing land types consist of local suitability, resistance, competitive advantage, neighborhood effects, and restricted conditions. Local suitability refers to the likelihood of each cell changing to each land system type, driven by driving factors. Resistance refers to the difficulty of changing to other land types. The competitive advantage determines whether the area of each land system type should increase or decrease to meet the demands. The neighborhood effect reflects how surrounding land types influence the central land type. Restricted conditions determine which locations and land types are not allowed to change. In each iteration, the first step for each cell is to check whether the land system type in that cell is allowed to change based on the restricted conditions. Next, the model calculates the sum of local suitability, resistance, competitive advantages, and neighborhood effects to determine the transition potential for each land system type. Finally, the land system type of the cell will be changed to the land type with the maximum transition potential. The detailed formulas are in Eqs. (1, 2).

1 $${\rm{T}}\left(c,t,i\right)=\left\{\begin{array}{ll}{\rm{T}}\left(c,t,0\right) & c\in \Phi \\ \left\{{k|}{P}_{c,k}={\max }_{j}{(P}_{c,1},{P}_{c,2},\cdots ,{P}_{c,j},\cdots {P}_{c,n},)\right\} & \left\{\begin{array}{l}{Con}(T(c,t,i),k)=1\\ c\notin \Phi \end{array}\right.\\ {\rm{T}}\left(c,t,i-1\right) & {else}\end{array}\right.$$

2 $${P}_{c,j}=\left\{\begin{array}{cc}{P{\rm{\_}}{loc}}_{c,j}+{P{\rm{\_}}{res}}_{{\rm{T}}\left(c,t,0\right)}+{P{\rm{\_}}{nei}}_{c,{\rm{T}}(c,t,0)}+{P{\rm{\_}}{cmp}}_{{\rm{T}}(c,t,0),j,(t,i)} & {if}{\rm{T}}\left(c,t,0\right)=j\\ {P{\rm{\_}}{loc}}_{c,j}+{P{\rm{\_}}{nei}}_{c,{\rm{T}}(c,t,0)}+{P{\rm{\_}}{cmp}}_{{\rm{T}}(c,t,0),j,(t,i)} & {if}{\rm{T}}\left(c,t,0\right)\ne j\end{array}\right.$$

Here, T(*c,t,i*) refers to the land type during the *i*-th iteration at time *t* at cell *c*. *P*_*c,j*_ refers to the transition potential for land type *j* and cell *c*. ${{P\_loc}}_{c,j}$ refers to the local suitability for land type *j* and cell *c*. ${{P\_}{res}}_{{\rm{T}}\left(c,t,0\right)}$ refers to the resistance for land type T(*c,t*,0). ${{P\_nei}}_{c,{\rm{T}}(c,t,0)}$ refers to the neighborhood effects for cell c and land type T(*c,t*,0). ${{P\_}{cmp}}_{{\rm{T}}(c,t,0),j,(t,i)}$ refers to the competitive advantage for changing land type T(*c,t*,0) to land type *j* during the *i*-th iteration at time *t*.

There are four improvements in the Land-N2N model compared with the original CLUMondo model^19^. First, the calculation of competitive advantages has been improved. In the original CLUMondo model, competitive advantages were calculated by assigning a supply capacity level to each land system type for each demand (Eq.(3)). This calculation cannot accurately capture demand changes resulting from changes in land system types. Therefore, in the Land-N2N model, competitive advantages are directly calculated on the basis of supply capacities. The calculation of improved competitive advantages is shown in Eq.(4). Second, the iteration mechanism has been improved. The improved iteration mechanism combines both finer and coarser-grained iterations, enhancing the ability to generate simulation results. The coarser-grained iterations aim to find results quickly. If the coarser-grained iterations fail to generate simulation results, finer-grained iterations are executed to generate simulation results on a finer scale. In the coarser-grained iterations, land system types can be changed simultaneously. In the finer-grained iterations, land system types can be changed one by one. Third, local suitability in the Land-N2N model is calculated using a random forest model instead of the logistic model. The random forest model is an algorithm for classification and regression^19,35,36^. In the Land-N2N model, the random forest model is used to calculate local suitability by establishing relationships between land types and driving factors. Fourth, the Land-N2N model proposed a useful traversing strategy to decrease the simulation time.

3 $$\left\{\begin{array}{l}{P{\rm{\_}}{cmp}}_{T(c,t,0),j,t}={\sum }_{d}{P{\rm{\_}}{cmp}}_{T(c,t,0),j,t,d}\\ {P{\rm{\_}}{cmp}}_{T(c,t,0),j,t,d}={sign}({L}_{j,d}-{L}_{T(c,t,0),d})\cdot {\omega }_{d}\cdot {{diff}}_{d,\left(t,i\right)}\\ \begin{array}{l}{{diff}}_{d,\left(t,i\right)}=\left\{\begin{array}{ll}0 & i=1\\ {{diff}}_{d,(t,i-1)}-\left(\frac{{{Supply}}_{d,\left(t,i-1\right)}-{{Demand}}_{d,t}}{{{Demand}}_{d,t}}\right)/\left({{Speed}}_{i}\times {R}_{t}\right) & i\ge 2\end{array}\right.\\ {{Speed}}_{i}=\left\{\begin{array}{ll}0.05 & i=1\\ {{Speed}}_{i-1}+0.0002 & i\ge 2\end{array}\right.\\ {sign}({L}_{j,d}-{L}_{T(c,t,0),d})=\left\{\begin{array}{ll}1, & {L}_{j,d} > {L}_{T(c,t,0),d}\\ 0, & {L}_{j,d}={L}_{T(c,t,0),d}\\ -1, & {L}_{j,d} < {L}_{T(c,t,0),d}\end{array}\right.\end{array}\end{array}\right.$$

4 $$\left\{\begin{array}{l}{P{\rm{\_}}{cmp}}_{{\rm{T}}(c,t,0),j,t}=\sum _{d}{{intertia}}_{d,(t,i)}\times \frac{{{CA}}_{{\rm{T}}(c,t,0),d}-{{CA}}_{j,d}}{\sum _{j}{{CA}}_{j,d}}\\ {{intertia}}_{d,(t,i)}=\left\{\begin{array}{ll}0 & i=1\\ {{intertia}}_{d,(t,i)}+\frac{{{Demand}}_{d,t}-{{Supply}}_{d,i-1}}{{{Speed}}_{i}} & i\ge 2\end{array}\right.\\ {{Speed}}_{i}=\left\{\begin{array}{ll}{Seed} & i=1\\ {{Speed}}_{i-1}+{Step} & i\ge 2\end{array}\right.\end{array}\right.$$

In Eqs. (3, 4), ${{P\_cmp}}_{{\rm{T}}(c,t,0),j,t}$ refers to the competitive advantage of changing land type T(*c,t*,0) to land type *j* during the *i*-th iteration at time *t*. ${{P\_cmp}}_{{\rm{T}}(c,t,0),j,t,d}$ refers to the competitive advantage for changing land type T(*c,t*,0) to land type *j* during the *i*-th iteration at time *t* for the *d*-th demand. *L*_*j,d*_ and ${L}_{T(c,t,0),d}$ refers to the conversion order for land type *j* and land type T(*c,t*,0). The conversion oder refers to the level of supply capacity. The conversion orders are all integers. ${{CA}}_{{\rm{T}}(c,t,0),d}$ and *CA*_*j,d*_ refers to the supply capacities of land type T(*c,t*,0) and land type *j*, respectively, to provide to *d*-th demand. *Demand*_*d,t*_ refers to the *d*-th demand at time *t*. *Supply*_*d,(t, i-1)*_ refers to the supply capacity provided by the land at the *i*-1-th iteration. *Speed*_*i*_ refers to an iteration parameter. *Seed* refers to the initial value of the iteration parameter. *Step* refers to the step size of the iteration parameter.

**Fig. 4:**
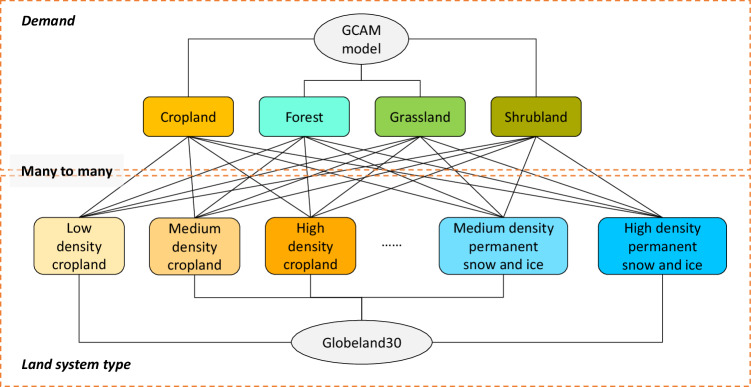
Many-to-many relationships between land system types and demands.
- Restricted conditions
  In this study, we specified which land system types are allowed to change. Generally, if a change between two land system types occurred from 2010 to 2020, we allowed the same change from 2020 to 2100. If the calculated restricted conditions prevent future land system changes, we need to adjust the restricted conditions. For example, if forest systems remained unchanged from 2010 to 2020, the calculated restricted conditions would prevent any future forest expansion. However, according to the GCAM demand in 2100, forest demand will be increased. In this case, we must adjust the calculated restricted conditions to align with the demands in the GCAM. Specifically, we did not allow for low, medium, and high artificial surface system types to be changed into other land system types because the GCAM currently assumes that the urban areas will remain unchanged until 2100.
- Resistance

To forecast land system changes by 2100, we calculated the necessary parameters for each basin in the Land-N2N model, including local suitability, supply capacity, restricted conditions, and resistance. The calculation methods are as follows:

- Local suitability
  We established a random forest model to calculate local suitability maps for each basin. The random forest regression model is shown in Eq. (5). To establish the random forest model, we prepared samples for each land system type, consisting of positive and negative samples. If the land type changes from *i* to *j*, we labeled the cell as the positive sample by generating a vector that consists of the label “1” and its corresponding driving factors. Other cells were labeled negative samples by generating a vector with the label “0” and its corresponding driving factors. In this study, we did not exclude the cells where the land system type remains unchanged and their corresponding driving factors from the negative samples. According to our experiments (Text S1), excluding these cells leads to a decrease in the accuracy of local suitability. The experiment results are shown in Table S3.
  5 $${P{\rm{\_}}{loc}}_{c,j}={RF}({X}_{1,c},{X}_{2,c},{\ldots ,X}_{m,c})$$
  Here, ${X}_{1,c},{X}_{2,c},{\ldots ,X}_{m,c}$ are driving factors of cell *c*.
- Supply capacity
  The supply capacity refers to the amount of demand that each land system type can fulfill for each demand. In this study, we established many-to-many relationships between land system types and demands, indicating that a land system type can fulfill multiple demands (Fig. 4). The process for calculating supply capacities consists of six steps. First, we assumed that the GCAM demands, namely areas of cropland, forest, grassland, and shrubland, are served by the corresponding 30 m resolution cropland, forest, grassland, and shrubland cells in Globeland30. Second, we calculated how much GCAM demands each 30 m resolution cell of cropland, forest, grassland, and shrubland can serve. Third, we overlay the 30 m resolution Globeland30 with the upscaled 990 m land system map, enabling us to calculate how many 30 m resolution cropland, forest, grassland, and shrubland cells are contained within each land system cell. Fourth, for each land system type, we calculated the average number of 30 m resolution cropland, forest, grassland, and shrubland cells within each land system cell. Fifth, by multiplying the average number of 30 m resolution cropland, forest, grassland, and shrubland cells (calculated in the fourth step) by their corresponding demand capacities (calculated in the second step), we obtained the supply capacities for each land system type. Sixth, the supply capacities obtained from the previous steps represent the GCAM demands served by each 990 m resolution land system cell. We then convert them to the supply capacities at a 1 km resolution to obtain the final supply capacity.

Resistance reflects the difficulty of changing a land system type. We calculated the proportion of each land system type that remained unchanged from 2010 to 2020 as the resistance for each basin. Similar to the restricted conditions, if the calculated resistances prevent future land system changes, we need to adjust the resistances. For example, if forest systems remained from 2010 to 2020, the calculated high resistance would prevent any future forest expansion. However, according to the GCAM demands in 2100, forest demand will be increased. Therefore, we need to adjust the calculated resistance to meet the demands in the GCAM.

## Data Records

**Fig. 5:**
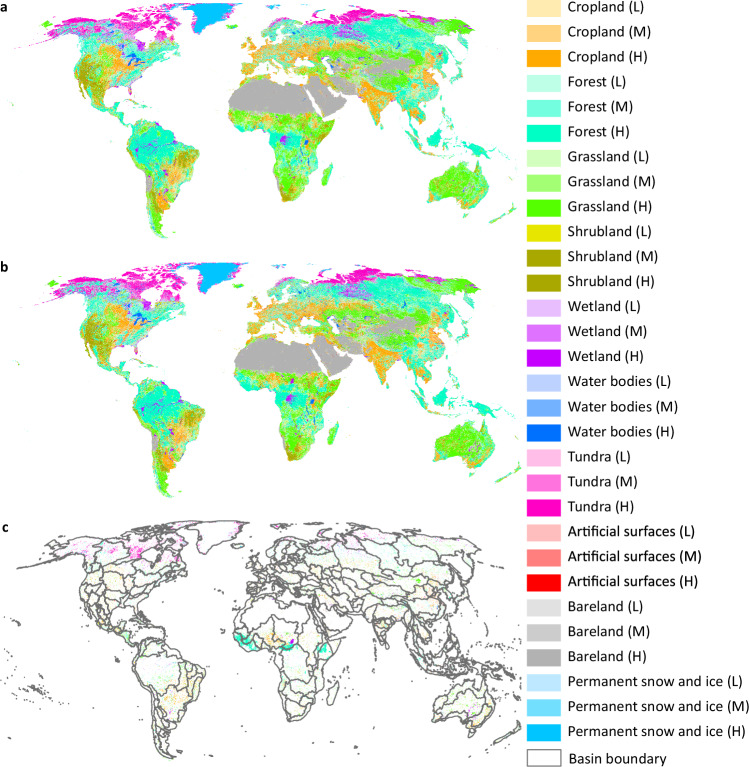
Land system maps for 2010 and 2020, along with the changes from 2010 to 2020. (**a**) Land system map for 2010. (**b**) Land system map for 2020. (**c**) Comparison map between the 2010 and 2020 land system maps. The blank areas indicate regions where the land system type remained unchanged from 2010 to 2020. The colorful areas indicate land system types in 2020 where changes occurred from 2010 to 2020.

**Fig. 6:**
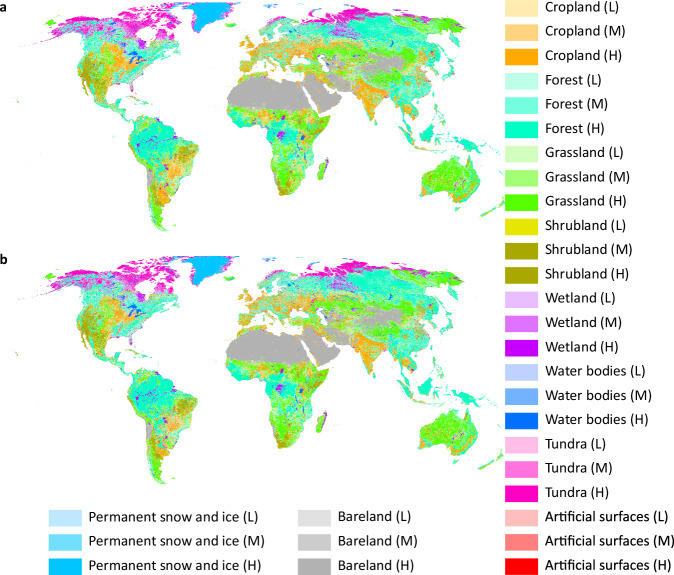
Land system maps for 2100. (**a**) Land system map for 2100 under the baseline scenario. (**b**) Land system map for 2100 under the 1.5 °C climate scenario.

**Fig. 7:**
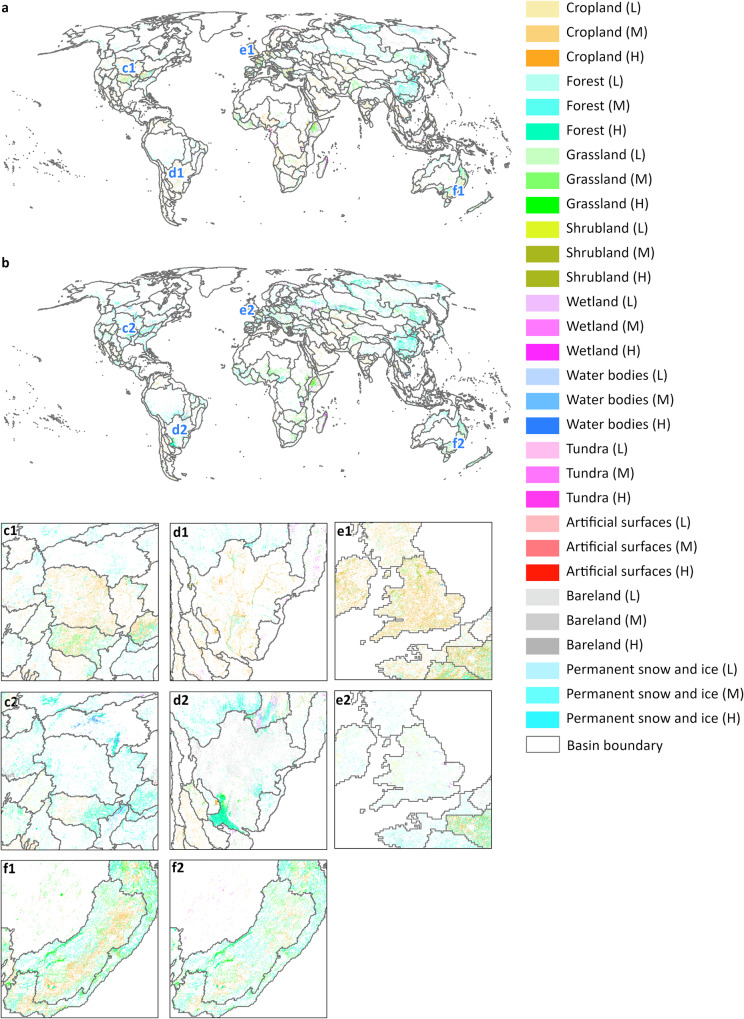
Comparison maps between the 2100 land system maps and 2020 land system maps. (**a**) Comparison map between the 2100 land system map under the baseline scenario and the 2020 land system map. The blank areas indicate the regions where the land system type remained unchanged from 2020 to 2100 under the baseline scenario. The colorful areas represent 2100 land system types where the land system type changed from 2020 to 2100 under the baseline scenario. (**b**) Comparison between the 2100 land system map under the 1.5 °C scenario and the 2020 land system map. The colorful and blank areas have the same meaning as those in (a). (**c1**), (**d1**), (**e1**), (**f1**). 2100 land system map of partial regions under the baseline scenario. (**c2**), (**d2**), (**e2**), (**f2**). 2100 land system map of partial regions under the 1.5 °C climate scenario.

The dataset is available at Figshare^37^. The dataset consists of 1 km global land system maps for 2010, 2020 (Fig. 5), and 2100 (Figs. 6, 7) under the baseline scenario and the 1.5 °C climate scenario^37^. These land system maps are stored in GeoTIFF format (.tif) and can be visualized using ArcGIS, ArcGIS Pro, or QGIS. We projected these land system maps into the “Cylindrical Equal Area (world)” reference system. The file names of the land system maps are listed in Table 3. In these maps, the integers represent different land system types (Table 4). In addition, we provide a color file (named “land system color.clr”) to assist users with visualization in ArcGIS or ArcGIS Pro.

**Table 3:** File names of the land system maps.

File name	Description
LS_2010.tif	2010 land system map
LS_2020.tif	2020 land system map
LS_2100_BA.tif	2100 land system map under the baseline scenario
LS_2100_15.tif	2100 land system map under the 1.5 °C climate scenario

**Table 4:** Integers and corresponding land system types.

No.	Land system type
0	Low density cropland
1	Medium density cropland
2	High density cropland
3	Low density forest
4	Medium density forest
5	High density forest
6	Low density grassland
7	Medium density grassland
8	High density grassland
9	Low density shrubland
10	Medium density shrubland
11	High density shrubland
12	Low density wetland
13	Medium density wetland
14	High density wetland
15	Low density water bodies
16	Medium density water bodies
17	High density water bodies
18	Low density tundra
19	Medium density tundra
20	High density tundra
21	Low density artificial surfaces
22	Medium density artificial surfaces
23	High density artificial surfaces
24	Low density bareland
25	Medium density bareland
26	High density bareland
27	Low density permanent snow and ice
28	Medium density permanent snow and ice
29	High density permanent snow and ice

## Technical Validation

### Accuracy evaluations for the Land-N2N model

In this study, we evaluated the accuracy of the Land-N2N model by simulating land system changes from 2010 to 2020. The key assumption is that if the model accurately simulates land system changes from 2010 to 2020, it can reliably be used to forecast future land system type changes. To quantify the evaluation, we selected the kappa coefficient^26^ and FoM^11^. The kappa coefficient was used to evaluate the similarity between the simulated and the actual land system maps. The FoM captures the accuracy of the simulated changes. The equations of the kappa coefficient and FoM are shown in Eqs. (6, 7), respectively. The value of the kappa coefficient ranges from −1 to 1^38^. A value greater than 0 indicates that the simulation accuracy is better than random, whereas a value less than 0 indicates that the simulation accuracy is worse than random. The value of the FoM ranges from 0 to 1. A larger FoM value indicates greater accuracy in detecting changes.

6 $${\rm{Kappa}}=\frac{{p}_{0}-{p}_{e}}{1-{p}_{e}}$$

7 $${\rm{Fom}}=\frac{{\rm{Hits}}}{{\rm{Hits}}+{\rm{Miss}}+{\rm{False\; alarm}}+{\rm{Wrong\; hits}}}$$

Wherein, *p*_0_ refers to the agreement between the simulated 2020 land system map and the actual 2020 land system map. *p*_*e*_ refers to the agreement due to chance. False alarm refers to the number of land cells that remained unchanged in the actual land system changes but changed in the simulated land system changes. Hits refers to the number of land cells that changed in the actual land system changes and correctly changed in the simulated land system changes. Miss refers to the number of land cells that changed in the actual land system changes but did not change in the simulated land system changes. Wrong hits refers to the number of land cells that changed in the actual land system changes but were incorrectly simulated as unchanged.

**Fig. 8:**
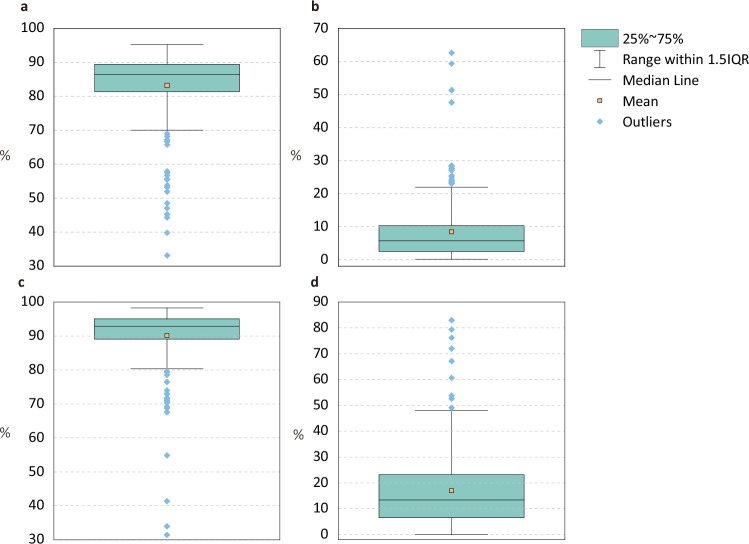
Box plots of the kappa coefficient and FoM for 235 water basins. (**a**) Box plot of the kappa coefficient for 30 land system types. (**b**) Box plot of FoM for 30 land system types. (**c**) Box plot of kappa coefficient for 10 land categories. (**d**) Box plot of FoM for 10 land categories.

**Fig. 9:**
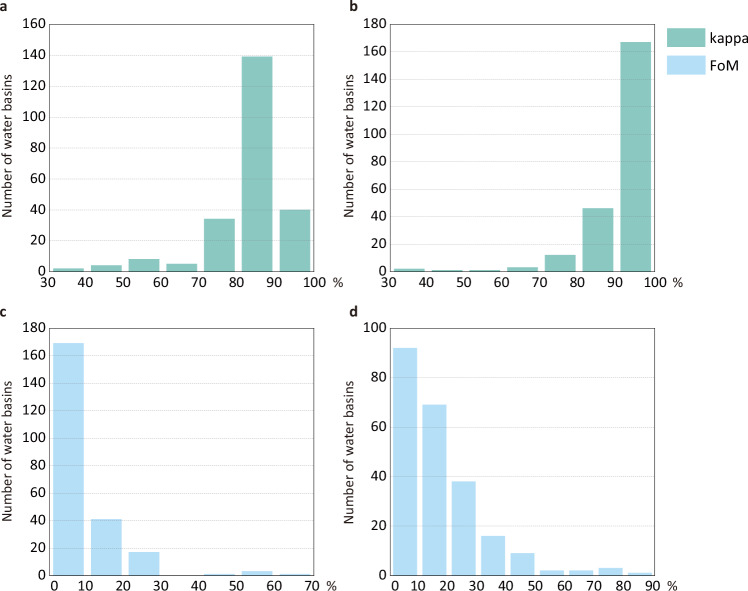
Histograms of kappa coefficient and FoM for 235 water basins. (**a**) Histogram of the kappa coefficient for 30 land system types. (**b**) Histogram of kappa coefficient for 10 land categories. (**c**) Histogram of FoM for 30 land system types. (**d**) Histogram of FoM for 10 land categories.

The evaluation results for each basin are shown in Fig. 8. The results are available at Figshare^37^. Specifically, we calculated the kappa coefficient and FoM values for the 30 land system types and 10 land categories (aggregating the low, medium, high density land system type for each land system). Regarding 30 land system types, the average kappa coefficient for 235 water basins was 83.14%, and the average FoM was 8.48%. Regarding 10 land categories, the average kappa coefficient for 235 water basins was 90.10%, and the average was 16.94%. Based on the kappa coefficient and FoM value, the Land-N2N model is able to simulate global land system changes effectively. In addition, the distribution of the kappa coefficient and FoM is shown in Fig. 9. For the 30 land system types, the kappa coefficient exceeds 80% for 179 water basins (76%). The FoM exceeds 10% for 63 water basins (27%). For the 10 land categories, the kappa coefficient exceeds 80% for 213 water basins. The FoM exceeds 10% for 140 water basins (60%).

**Fig. 10:**
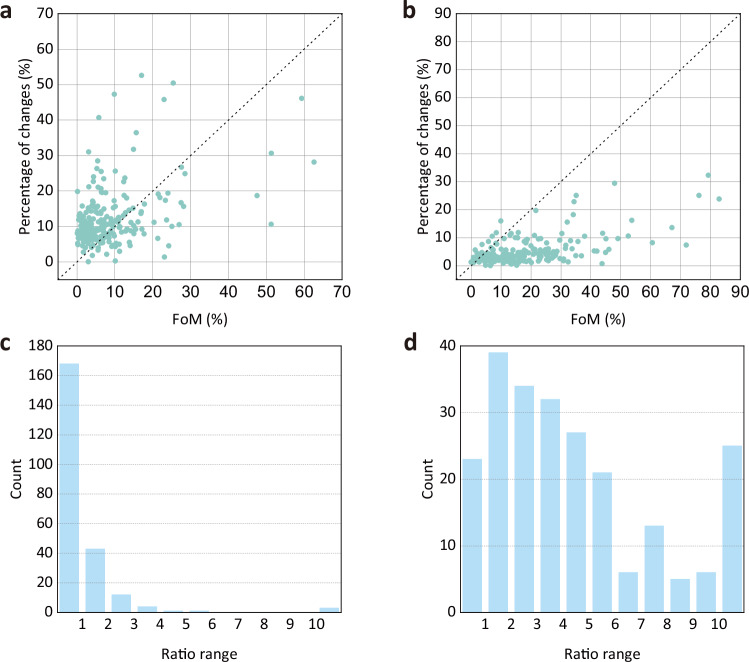
Ratios of actual land changes and the FoM for 235 water basins. (**a**) Scatter plot for 30 land system types. (**b**) Scatter plot for 10 land categories. (**c**) Histogram of actual land changes and the FoM for 30 land system types. (**d**) Histogram of actual land changes and the FoM for 10 land categories. In panels (**c,d**), the rightmost bar represents the number of water basins where the ratio exceeds.

To comprehensively evaluate the model performance, we also calculated the percentages of actual land changes from 2010 to 2020. And we compared these percentages with the FoM (Fig. 10). The calculated results are available at Figshare^37^. For 30 land system types, the average percentage of actual land changes from 2010 to 2020 was 12.12%. The average ratio of the FoM to the percentages of actual land changes was 1.48. For 10 land categories, the average percentage of actual land changes from 2010 to 2020 was 4.90%. The average ratio of the FoM to the percentages of actual land changes was 9.46. In Fig. 10, we found the values of FoM did not align perfectly with the percentages of actual land changes, which demonstrates the reliability of FoM.

### Comparison with accuracy evaluations using the original CLUMondo model

Compared with the original CLUMondo model, the Land-N2N model simulated land system changes more accurately. Specifically, we randomly selected four water basins to simulate land system changes using both the original CLUMondo model and the Land-N2N model. Table 5 shows the kappa coefficient and FoM. In the Mediterranean Sea East Coast and Papua New Guinea Coast, the kappa coefficient and FoM using the Land-N2N model were average 32.15% and 290.50% higher than the kappa coefficient and FoM using the original CLUMondo model, respectively. In the Guadalquivir and Chao Phraya, the original CLUMondo model did not generate simulation results.

**Table 5:** Kappa coefficient and FoM for randomly selected water basins using the original CLUMondo model and the Land-N2N model.

Water basin	Original CLUMondo model		Land-N2N model	
	Kappa	FoM	Kappa	FoM
Mediterranean Sea East Coast	51.46%	3.33%	81.88%	10.14%
Guadalquivir	—	—	91.75%	4.99%
Chao Phraya	—	—	57.89%	3.10%
Papua New Guinea Coast	82.25%	2.17%	86.51%	10.34%

Note: “-” indicates that no simulation result was generated for the water basin using the original CLUMondo model.

### Comparison with other datasets

Compared with evaluations of other land datasets, the accuracy of the kappa coefficient and FoM demonstrated that the Land-N2N model effectively simulated global land system changes. Table 6 shows the accuracy of other similar datasets. Specifically, the datasets reported by Li, *et al*.^11^ were only evaluated with the FoM for 17 regions worldwide. The maximum FoM value in our evaluations exceeded that of this dataset. Although the kappa coefficient and FoM reported by Zhang, *et al*.^10^ are higher than ours, their thematic resolution is lower than ours. Thematic resolution refers to the number of categories used to classify land types^39,40^. In our study, the thematic resolution consists of 30 land system types. In addition, the kappa coefficient of the datasets of Luo, *et al*.^17^ and Dong, *et al*.^24^ is lower than that of our dataset.

**Table 6:** Accuracy of other datasets.

Dataset	Resolution	Thematic resolution	Scenario	Study area	Kappa	FoM
Li, *et al*.^11^	1 km	6	IPCC SRES	Global	—	10.11% ~ 28.98%
Zhang, *et al*.^10^	1 km	6	SSP-RCP	Global	94%	10%
Luo, *et al*.^17^	1 km	5	SSP-RCP	China	66%	—
Dong, *et al*.^24^	1 km	6	SSP-RCP	China	79%	18.92%
Chen, *et al*.^44^	1 km	20	SSP-RCP	Global	86.4%	10.2%

Note: “-” indicates that the dataset did not use the kappa coefficient or FoM for accuracy evaluation of the dataset. The decimals in the table are consistent with those reported in the original literature.

## Usage Notes

In this study, we generated a dataset consisting of 2010, 2020, and 2100 land system maps under the baseline scenario and the 1.5 °C climate scenario at 1 km resolution. According to the existing research, the 1 km resolution is the finest to simulate land change simulations^11,41–43^. Compared with other datasets, there are three key improvements. First, we used the Land-N2N model to simulate land system changes. Our experiments revealed that the Land-N2N model simulated land system changes more accurately than the original CLUMondo model. In addition, the Land-N2N model makes it easier to obtain simulation results. Second, we forecasted future land system changes under climate pledges. Simulating land system changes under climate pledges is able to help us understand the potential impacts of future land changes and provide insights for adjusting these climate pledges. Third, we established land systems using an upscaling method. Compared with traditional land use or land cover, land systems provide more detailed information, such as local density.

Our datasets reveal the changes in 27 land system types worldwide (detailed information can be accessed in the supplementary table). Under the 1.5 °C climate scenario, more than 15% of five land system types change to other land system types, namely low density cropland (21%), medium density cropland (26%), low density wetland (17%), low density permanent snow and ice (28%), and medium density permanent snow and ice (21%). Under the baseline scenario, more than 15% of six land system types change to other land system types, namely low density cropland (16%), low density wetland (18%), medium density wetland (16%), low density water bodies (21%), medium density water bodies (16%), and low density permanent snow and ice (29%). Regarding the changes between land system types, 10% of low density cropland, 10% of medium density cropland, and 7% of high density cropland will be changed into high density forest under the 1.5 °C climate scenario (Fig. S1). Additionally, under the baseline scenario, high density cropland and high density forest will increase by 6% and 14%, respectively. Specifically, the 13,106 km^2^ (16%) low density cropland will be changed to high density cropland, and 317,404 km^2^ (16%) medium density cropland will be changed to high density forest (Fig. S2).

Our dataset provides valuable data support for land management and other research. In terms of land management, the 2100 land system maps under the baseline scenario and the 1.5 °C climate scenario can be used to compare the impacts of different climate pledges on land systems. In terms of other research applications, our datasets can serve as foundational data. For example, the land system maps can be used to assess changes in terrestrial ecosystem carbon storage^18,23^. In addition, the land system maps can be used to explore the risks of sea level rise.

When using land system maps, it is important to note that our datasets focus on vegetation and agricultural land system changes rather than changes in artificial surface systems. This occurs because the GCAM currently assumes the urban remains unchanged in the future. As a result, the dataset is not suitable for research related to urban expansion. We acknowledged that this assumption is a limitation. However, given the global proportion of artificial surface systems, we believe this limitation is acceptable. Specifically, artificial surface systems accounted for just 0.66% in 2010 and 0.85% in 2020.

Additionally, in the study, we only used static driving factors due to a lack of data. Here, static driving factors refer to those that remain unchanged over time. In contrast, dynamic driving factors are those that change over time. In our future work, we will consider trying to use dynamic driving factors to calculate local suitability.

## Supplementary information

Supplementary Information of Global land system maps at 1 km resolution for 1.5 °C climate

Supplementary table

## Data Availability

In this study, we used the codes submitted by Gao, *et al*.^19^. These codes can be accessed at https://github.com/gaoyifan2021/Land-N2N-v1.
