# Supplementary material for: Global land system maps at 1 km resolution for 1.5 °C climate
Source: Sci Data. 2025 Apr 22;12:672. doi: 10.1038/s41597-025-04991-0 (PMC12015323; doi:10.1038/s41597-025-04991-0)
Supplement: Supplementary file 1 — Supplementary Information of Global land system maps at 1 km resolution for 1.5 °C climate [file 41597_2025_4991_MOESM1_ESM.docx]

**Supplementary Information of**

***Global land system maps at 1 km resolution for* *1.5 °C climate***

Yifan Gao^1^, Haewon McJeon^2^, Yang Ou^3,4^, Li Chen^1^, Jiaying Lv^1^, Delin Fang^1^, Yuanhui Wang^1^, Sijing Ye^1^, Changqing Song^1^, Peichao Gao^1,^*

1. State Key Laboratory of Earth Surface Processes and Hazards Risk Governance, Faculty of Geographical Science, Beijing Normal University, Beijing 100875, China

2. Graduate School of Green Growth and Sustainability, Korea Advanced Institute of Science and Technology, Republic of Korea

3. College of Environmental Sciences and Engineering, Peking University, Beijing, China

4. Institute of Carbon Neutrality, Peking University, Beijing, China

*Corresponding author(s): Peichao Gao (gaopc@bnu.edu.cn)

**Content**

[Text 2](#_Toc194933475)

[Text S1 Comparison of sample selection strategies. 2](#_Toc194933476)

[Tables 3](#_Toc194933477)

[Table S1 Dataset materials. 3](#_Toc194933478)

[Table S2 Demands in 2020 and 2100 (Unit: km^2^). 5](#_Toc194933479)

[Table S3. AUC in Strategy A and Strategy B for 11 water basins. 6](#_Toc194933480)

[Figures 7](#_Toc194933481)

[Figure S1 Summary of the cell-by-cell transformations of global land systems from 2020 to 2100 under the 1.5 °C climate pledge scenario 7](#_Toc194933482)

[Figure S2 Summary of the cell-by-cell transformations of global land systems from 2020 to 2100 under the baseline scenario 8](#_Toc194933483)

[References 9](#_Toc194933484)

# Text

## Text S1 Comparison of sample selection strategies.

In this study, we set comparison experiments for two different sample selection strategies, namely Strategy A and Strategy B. Strategy A includes the cells where land type remained unchanged in negative samples. Strategy B excludes the cells where land type remained unchanged in negative samples. We randomly selected 11 water basins as the study areas. We used the Area Under the Curve (AUC) to evaluate the performance of the two strategies. A higher AUC indicates greater accuracy of the sample selection strategies. For the two strategies, aside from the different negative sample selection strategies, all other settings and parameters remain the same.

# Tables

## Table S1 Dataset materials.

| Category | | Data | Source |
| --- | --- | --- | --- |
| Land cover | | Globeland30 | Chen, et al. ^1^ |
| Driving factors | Soil | Bulk density | Hengl, et al. ^2^ |
|  |  | Cation exchange capacity |  |
|  |  | Clay content |  |
|  |  | Coarse fragments volumetric |  |
|  |  | Derived available soil water capacity |  |
|  |  | Organic carbon density |  |
|  |  | PH in H_2_O |  |
|  |  | Sand content |  |
|  |  | Silt content |  |
|  |  | Texture class |  |
|  | Socio-economic | Market access index | Verburg, et al. ^3^ |
|  |  | Market influence index ($/person) |  |
|  |  | Market density index |  |
|  |  | Nighttime lights | DMSP-OLS Nighttime Lights Time Series Version 4 (https://datadryad.org/stash/dataset/doi:10.5061/dryad.dk1j0) |
|  |  | Total GDP (PPP, purchasing power parity) | Kummu, et al. ^4^ |
|  |  | Gridded Population of the World (GPW) | NASA Socioeconomic Data and Applications Center (SEDAC) () |
|  | Accessibility | Time to nearest cities | Weiss, et al. ^5^ |
|  |  | Rivers and lake centerlines | Natural Earth (https://www.naturalearthdata.com/) |
|  |  | Distance to the nearest railway |  |
|  |  | Railroads |  |
|  |  | Travel time one meter (motorized) | Weiss, et al. ^6^ |
|  |  | Travel time one meter (walking-only) |  |
|  |  | Time to nearest healthcare facility (motorized) |  |
|  |  | Time to nearest healthcare facility (walking-only) |  |
|  | Agriculture and Vegetation | 175 Crops Yield per hectare | Monfreda, et al. ^7^ |
|  |  | Gross primary production-March | Wang and Zhang ^8^ |
|  |  | Gross primary production-June |  |
|  |  | Gross primary production-September |  |
|  |  | Gross primary production-December |  |
|  |  | NDVI-March | NASA EOSDIS Land Processes DAAC (https://land.copernicus.eu/global/) |
|  |  | NDVI- June |  |
|  |  | NDVI- September |  |
|  |  | NDVI- December |  |
|  | Terrain | Elevation | Fick and Hijmans ^9^ |
|  |  | variance of elevation | Calculated by elevation |
|  |  | Slope |  |
|  |  | Aspect |  |
|  | Climate | Annual mean precipitation | Hengl ^10^ |
|  |  | Mean precipitation-March |  |
|  |  | Mean precipitation-June |  |
|  |  | Mean precipitation-September |  |
|  |  | Mean precipitation-December |  |
|  |  | Annual mean temperature | Hengl ^11^ |
|  |  | Mean temperature-March |  |
|  |  | Mean temperature-June |  |
|  |  | Mean temperature-September |  |
|  |  | Mean temperature-December |  |
|  | Livestock | Buffaloes | Gridded Livestock of the World – 2010 (GLW 3) (https://dataverse.harvard.edu/dataverse/glw) |
|  |  | Cattle |  |
|  |  | Chickens |  |
|  |  | Ducks |  |
|  |  | Goats |  |
|  |  | Horses |  |
|  |  | Pigs |  |
|  |  | Sheep |  |
|  | Land cover density | Cropland density | Calculated by Globeland30^1^ |
|  |  | Forest density |  |
|  |  | Grassland density |  |
|  |  | Shrubland density |  |
|  |  | Wetland density |  |
|  |  | Water bodies density |  |
|  |  | Tundra density |  |
|  |  | Artificial surfaces density |  |
|  |  | Bareland density |  |
|  |  | Permanent ice and snow density |  |

## Table S2 Demands in 2020 and 2100 (Unit: km^2^).

| Demands | 2020 | 2100 under the 1.5 °C climate pledge scenario | 2100 under the baseline scenario |
| --- | --- | --- | --- |
| Cropland | 15,859,969 | 14,400,774 | 16,508,681 |
| Forest | 29,926,061 | 31,873,255 | 30,639,196 |
| Grassland | 52,463,815 | 52,246,034 | 51,468,774 |
| Shrubland | 11,797,005 | 11,526,781 | 11,430,198 |

## Table S3. AUC in Strategy A and Strategy B for 11 water basins.

| Basin | Average AUC in Strategy A | Average AUC in Strategy B |
| --- | --- | --- |
| Africa North West Coast | 0.94 | 0.37 |
| Australia South Coast | 0.92 | 0.50 |
| Bo Hai Korean Bay North Coast | 0.87 | 0.56 |
| Atlantic Ocean Seaboard | 0.89 | 0.53 |
| Douro | 0.76 | 0.48 |
| Gironde | 0.79 | 0.53 |
| Huang He | 0.94 | 0.53 |
| Java Timor | 0.83 | 0.54 |
| Lake Chad | 0.97 | 0.51 |
| Lower Mississippi River Basin | 0.82 | 0.50 |
| Poland Coast | 0.75 | 0.53 |

# Figures

## Figure S1 Summary of the cell-by-cell transformations of global land systems from 2020 to 2100 under the 1.5 °C climate pledge scenario


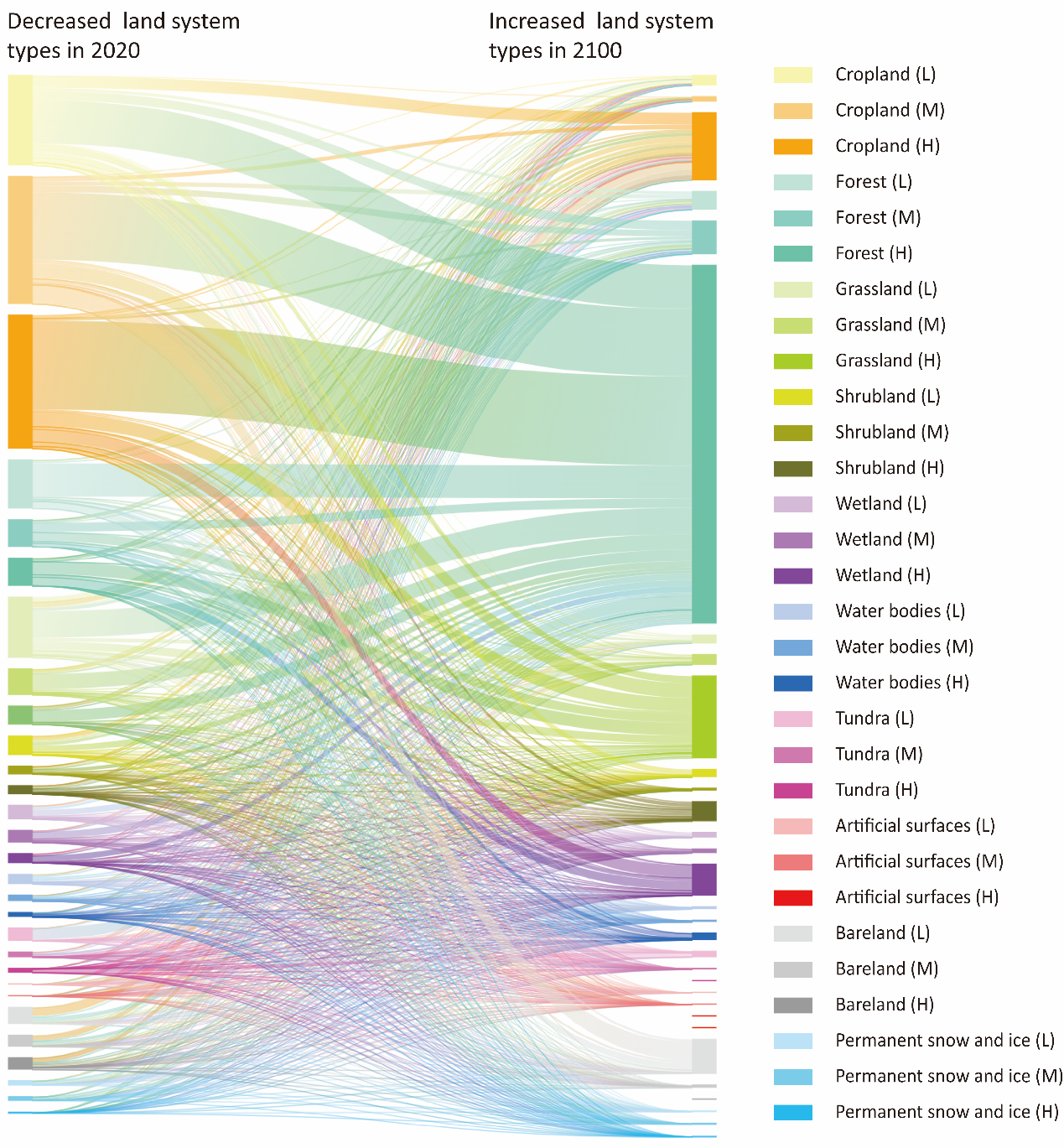


## Figure S2 Summary of the cell-by-cell transformations of global land systems from 2020 to 2100 under the baseline scenario


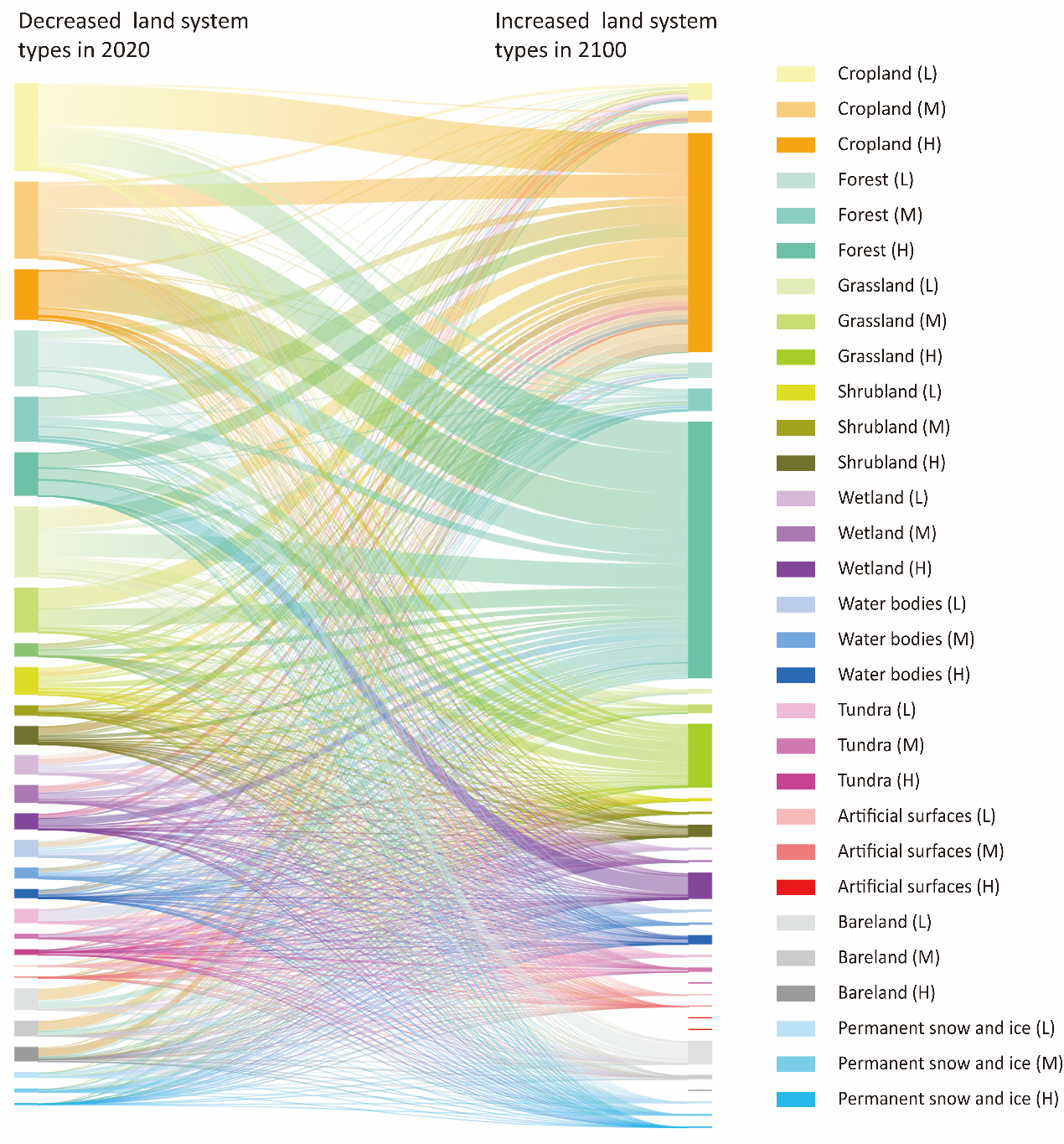


# References

1 Chen, J., Ban, Y. & Li, S. Open access to Earth land-cover map. *Nature* **514**, 434-434, doi:<https://doi.org/10.1038/514434c> (2014).

2 Hengl, T., Mendes de Jesus, J., Heuvelink, G. B., Ruiperez Gonzalez, M., Kilibarda, M., Blagotić, A., Shangguan, W., Wright, M. N., Geng, X. & Bauer-Marschallinger, B. SoilGrids250m: Global gridded soil information based on machine learning. *PLoS one* **12**, e0169748, doi:<https://doi.org/10.1371/journal.pone.0169748> (2017).

3 Verburg, P. H., Ellis, E. C. & Letourneau, A. A global assessment of market accessibility and market influence for global environmental change studies. *Environmental Research Letters* **6**, 034019, doi:<https://doi.org/10.1088/1748-9326/6/3/034019> (2011).

4 Kummu, M., Taka, M. & Guillaume, J. H. Gridded global datasets for gross domestic product and Human Development Index over 1990–2015. *Scientific data* **5**, 1-15, doi:<https://doi.org/10.1038/sdata.2018.4> (2018).

5 Weiss, D. J., Nelson, A., Gibson, H., Temperley, W., Peedell, S., Lieber, A., Hancher, M., Poyart, E., Belchior, S. & Fullman, N. A global map of travel time to cities to assess inequalities in accessibility in 2015. *Nature* **553**, 333-336, doi:<https://doi.org/10.1038/nature25181> (2018).

6 Weiss, D., Nelson, A., Vargas-Ruiz, C., Gligorić, K., Bavadekar, S., Gabrilovich, E., Bertozzi-Villa, A., Rozier, J., Gibson, H. & Shekel, T. Global maps of travel time to healthcare facilities. *Nature medicine* **26**, 1835-1838, doi:<https://doi.org/10.1038/s41591-020-1059-1> (2020).

7 Monfreda, C., Ramankutty, N. & Foley, J. A. Farming the planet: 2. Geographic distribution of crop areas, yields, physiological types, and net primary production in the year 2000. *Global biogeochemical cycles* **22**, doi:<https://doi.org/10.1029/2007GB002947> (2008).

8 Wang, S. & Zhang, Y. Global gross primary production dataset based on NIRv. *National Tibetan Plateau Data Center* doi:<https://doi.org/10.6084/m9.figshare.12981977.v2> (2020).

9 Fick, S. E. & Hijmans, R. J. WorldClim 2: new 1‐km spatial resolution climate surfaces for global land areas. *International journal of climatology* **37**, 4302-4315, doi: <https://doi.org/10.1002/joc.5086> (2017).

10 Hengl, T. Monthly precipitation in mm at 1 km resolution based on SM2RAIN-ASCAT 2007-2018, IMERGE, CHELSA Climate and WorldClim (Version 0.2). *Zenodo* doi:<http://doi.org/10.5281/zenodo.3256275> (2018).

11 Hengl, T. Long‐term MODIS LST day‐time and night‐time temperatures, sd and differences at 1 km based on the 2000–2017 time series. doi:<https://doi.org/10.5281/zenodo.6458406> (2018).
